# High mRNA expression level of IL-6R was associated with better prognosis for patients with ovarian cancer: a pooled meta-analysis

**DOI:** 10.1038/s41598-017-09333-8

**Published:** 2017-08-18

**Authors:** Qu Chen, Bin Xu, Lei Lan, Da Yang, Min Yang, Jingting Jiang, Binfeng Lu, Yueping Shen

**Affiliations:** 1 Jiangsu Key Laboratory of Preventive and Translational Medicine for Geriatric Diseases, Department of Epidemiology and Biostatistics, School of Public Health, Soochow University, Suzhou, Jiangsu Province China; 2 Department of Tumor Biological Treatment, the Third Affiliated Hospital of Soochow University, Changzhou, Jiangsu Province China; 3 Jiangsu Engineering Research Center for Tumor Immunotherapy, the Third Affiliated Hospital of Soochow University, Changzhou, Jiangsu Province China; 4 Department of Immunology, University of Pittsburgh School of Medicine, E1040 Biomedical Science Tower, 200 Lothrop Street, Pittsburgh, USA; 5 Center for Pharmacogenetics, Department of Pharmaceutical Sciences, University of Pittsburgh, Pittsburgh, USA

## Abstract

Interleukin-6 acts as both a pro-inflammatory cytokine and an anti-inflammatory myokine. IL-6/IL-6R signaling pathway, in particular, has been proposed to be a pivotal cytokine promoting ovarian cancer progression. This study aimed to elucidate potential clinical and biological function of IL-6R mRNA expression in ovarian cancer. We used the keywords “ovarian cancer” and searched through GEO database and finally a total of 7 studies together with TCGA database were incorporated in this analysis. We used *Cutoff Finder* to determine a cutoff point and stratified patients into two groups and found that high-expression of IL-6R mRNA in tumor tissues was a positive prognostic factor for overall survival. Simultaneously, high expression level of IL-6R mRNA correlates with better survival of patients who had additional chemotherapy treatment. These analyses suggested a possible role of tumoral expression of IL-6R in ovarian cancer. In conclusion, our results showed that mRNA levels of IL-6R in ovarian cancer was positively associated with better prognosis and sensitivity to chemotherapy and can potentially be used as a prognostic marker for this cancer.

## Introduction

Ovarian cancer is the leading lethal gynecological cancer worldwide. It accounts for approximately 200,000 new cases per year globally^1^. The poor prognosis is partly due to ovarian cancer usually does not show symptoms until it has been widespread^2^. In the past 20 years, the therapeutic effect of malignant epithelial ovarian tumor has not been improved, 5 year survival rate remained around 30~40%, and the mortality ranks first in gynecologic malignancies. Malignant epithelial ovarian tumor has become a serious threat to women’s life and health. The composition of ovarian tissue is very complex, which has the most organ types of primary tumors.

Tumor microenvironment (TME) is a pathological environment composed of tumor cells, stromal cells, cytokines and immune cells^3^. It has the characteristics of hypoxia, acidosis and interstitial hypertension^4^. Ovarian cancer is immunogenic tumor, using many immunosuppressive methods to evade immune elimination, and mainly spread through peritoneal implants and direct spread. Understanding cytokines, immune and inflammatory responses in the TME may be the key to understand the progression of ovarian cancer.

Recent data suggested that a broad spectrum of inflammatory factors are involved in the development and progression of ovarian cancer^5^. Hence, identifying new additional prognostic and predictive biomarkers may help identify high risk patients, predict outcome of ovarian cancer and even offer a therapeutic strategy. There are more than 16 different cytokines together with corresponding receptors expressed in normal ovaries^6^. And several particular cytokines and/or their receptors were expressed abnormally^7^. Interleukin-6(IL-6), in particular, has been proposed to be a pivotal cytokine promoting ovarian cancer progression. IL-6 signaling through Interleukin-6 receptor(IL-6R) can lead to cell survival, proliferation, angiogenesis, and confers resistance to apoptosis induced by conventional therapies through several pathways^8^. Antagonizing IL-6/IL-6R signaling was accepted to have therapeutic activity through inhibition of cytokine network in ovarian cancer cell^9^. Increased expression of IL-6R protein has been observed in ovarian cancer cell lines, cancer tissue, malignant ascites and serum^10–13^. Kim *et al*.^14^ demonstrated that IL-6 binding to IL-6R increases invasion in ascites. Isobe *et al*.^10^ demonstrated that high IL-6R protein expression in cancer tissue showed significantly worse progression free survival(PFS) than those who had low or negative expression. Therefore, blockade of anti- IL-6/IL-6R signaling has been proposed as a therapeutic approach for ovarian cancer^15, 16^. Indeed, Tocilizumab, an anti-IL-6R monoclonal antibody, in combination with chemotherapy, has shown an acceptable safety profile and a possible immunological benefit in patients with advanced ovarian cancer in a phase I trial^17^. However, clinical benefit for this approach has not been demonstrated. Despite evidences supported a possible protumor role of IL-6R in ovarian cancer, other studies suggested otherwise. Coward *et al*.^18^ have found no association between IL-6R protein expression and survival of patients with ovarian cancer. It has also been found that tumors with a high expression of IL-6R displayed a longer disease-specific survival (DSS), especially in late stage tumors^19^. Thus, the exact role of IL-6R during ovarian development has therefore not been resolved.

In the present study, we firstly analyzed the correlation between the IL-6R mRNA level and its prognostic value in ovarian cancer. We also tried to understand the potential effect of IL-6R on TME and the clinical significance in target-therapy.

## Results

### Study characteristics

**Figure 1:**
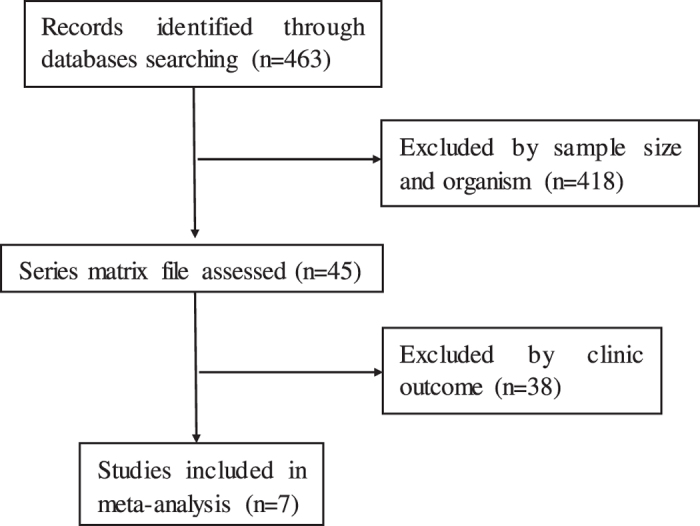
Flow diagram of dataset selection process.

A total of 7 related publications were identified from the NCBI Pubmed and Gene Expression Ominibus (GEO) database in NCBI (GSE9891^20^, GSE17260^21^, GSE26193^22^, GSE26712^23^, GSE32062^24^, GSE49997^25^, GSE63885^26^). Including TCGA, there were 8 datasets that be analyzed in this article. In the initial screening, a total of 463 potentially relevant datasets were selected for keyword retrieval. 45 datasets were retrieved after screening sample size and organism. After reading summary and the clinic outcome of those data, a total of 7 microarray data that met the inclusion criteria were included in the present study (Fig. 1). Table 1 showed the baseline characteristics of all included studies. Data of 1735 patients from Australia, Japan, France, America, Vienna, Poland and Singapore were included in this analysis. All of those data reported overall survival (OS), mRNA expression level of IL-6R.

**Table 1:** Baseline Characteristics of 8 Microarray Data for Pooled-Analysis.

GEO datasets	author	year	country	duration months(s) median(range)	sample size	stage	outcome measures	quality score
GSE9891	Tothill R.	2008	Australia	28 (1–214)	240	I/II/III/IV(11/10/197/21)	OS	8
GSE17260	Yoshihara K.	2010	Japan	30.50 (1–81)	110	III/IV(93/17)	OS	7
GSE26193	Mateescu B.	2011	France	36 (0.10–242.64)	107	I/II/III/IV(21/10/59/17)	OS	7
GSE26712	Birrer MJ.	2015	America	38.28 (0.72–163.80)	185	NR	OS	7
GSE32062	Gonzales KA.	2015	Singapore	41.50 (1–128)	260	III/IV(104/56)	OS	8
GSE49997	Pils D.	2014	Vienna	24.50 (1–49)	194	NR	OS	8
GSE63885	Lisowska K.	2014	Poland	36.83 (3.47–136.00)	75	II/III/IV(2/63/10)	OS	6
TCGA	TCGA	2015	America	28.67 (0.26–180.20)	564	I/II/III/IV(15/27/434/84)	OS	9

NR = not report; OS = overall survival.

The eight eligible datasets included in this meta-analysis have been performed a quality assessment according to Newcastle-Ottawa Quality Assessment Scale (NOS). The quality score span was from 6 to 9 and the mean score is 7.5. Thus, all of those eight studies were included in following analysis.

### Overall survival

**Figure 2:**
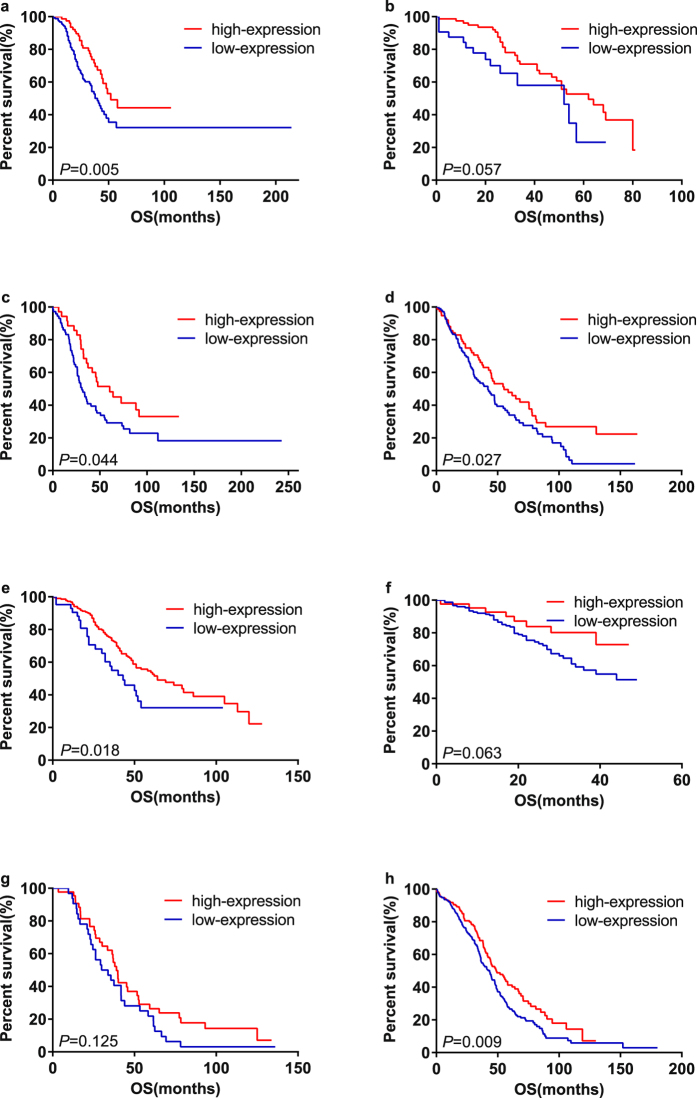
High mRNA expression level of IL-6R in ovarian cancer patients was associated with better prognosis. (**a**) Kaplan-Meier analysis of dataset GSE9891. (**b**) Kaplan-Meier analysis of dataset GSE17260.(**c**) Kaplan-Meier analysis of dataset GSE26193. (**d**) Kaplan-Meier analysis of dataset GSE26712. (**e**) Kaplan-Meier analysis of dataset GSE32062. (**f**) Kaplan-Meier analysis of dataset GSE49997. (**g**) Kaplan-Meier analysis of dataset GSE63885. (**h**) Ovarian cancer data from TCGA database.

**Figure 3:**
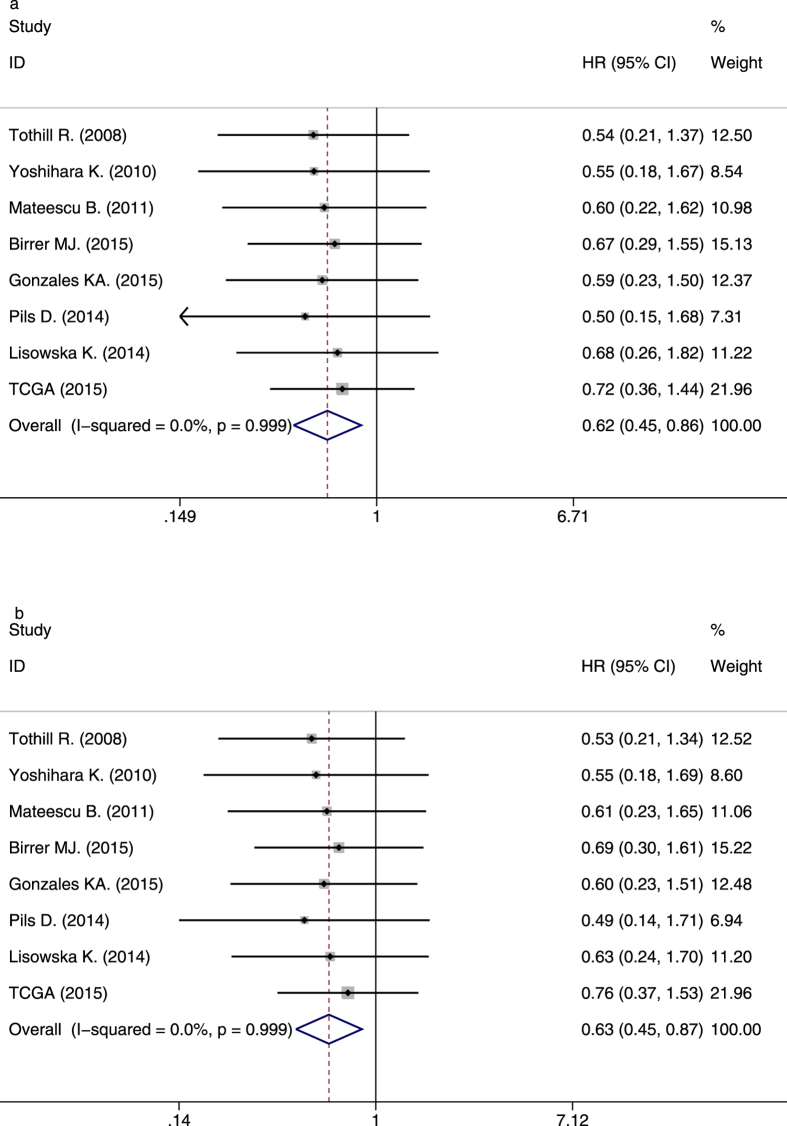
Forest plots of studies evaluating hazard ratios of high IL-6R mRNA expression in ovarian cancers for OS. (**a**) univariate analysis. (**b**) multivariate analysis.

Eight studies provided suitable data for OS analysis. Univariate analysis and multivariate analysis were respectively carried out for each article (Supplementary Table S1). *P* values, HRs and 95%CIs of IL-6R mRNA in each article were shown in Supplementary Table S2 and Fig. 2. As there was no obvious statistical heterogeneity in all of those 8 datasets both in univariate survival analysis and multivariate survival analysis (I^2^ = 0.0%, *P* = 0.999; I^2^ = 0.0%, *P* = 0.999), a fixed-effects model was used to calculate the pooled HR. Overall, this meta-analysis demonstrated that a higher expression of IL-6R mRNA was significantly associated with better OS (pooled HR = 0.62; 95% CI = 0.45–0.86; *P* = 0.004 in univariate analysis; pooled HR = 0.63; 95% CI = 0.45–0.86; *P* = 0.005 in multivariate analysis). The forest plots of study-specific HRs for OS were presented in Fig. 3.

**Figure 4:**
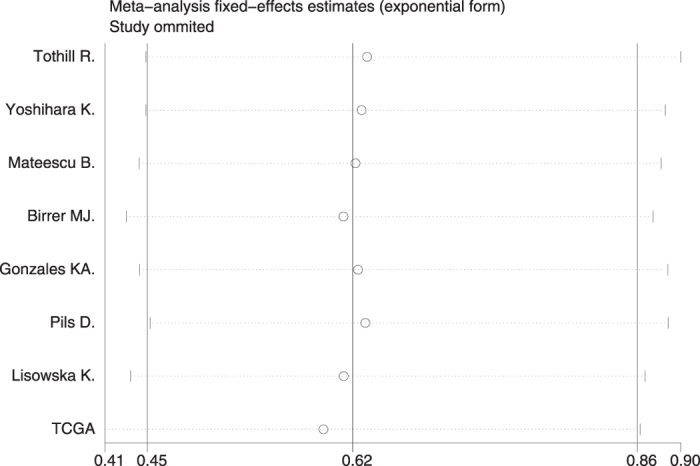
Sensitivity analysis on the relationships between IL-6R mRNA expression and overall survival in ovarian cancer patients.

We performed sensitivity analysis using the fixed-effects model by sequential excluding individual study, and it did not substantially change the results, indicating that the results were credible (Fig. 4).

Funnel plots, Egger’s and Begg’s tests were used to evaluate the publication bias of all 8 studies. Visual inspection of Begg’s funnel plot (Supplementary Fig. S1) indicated no evidence of significant publication bias. Begger’s test, *P* = 0.083; Egger’s test, *P* = 0.515.

### Stratification analysis of IL6R mRNA on survival

**Figure 5:**
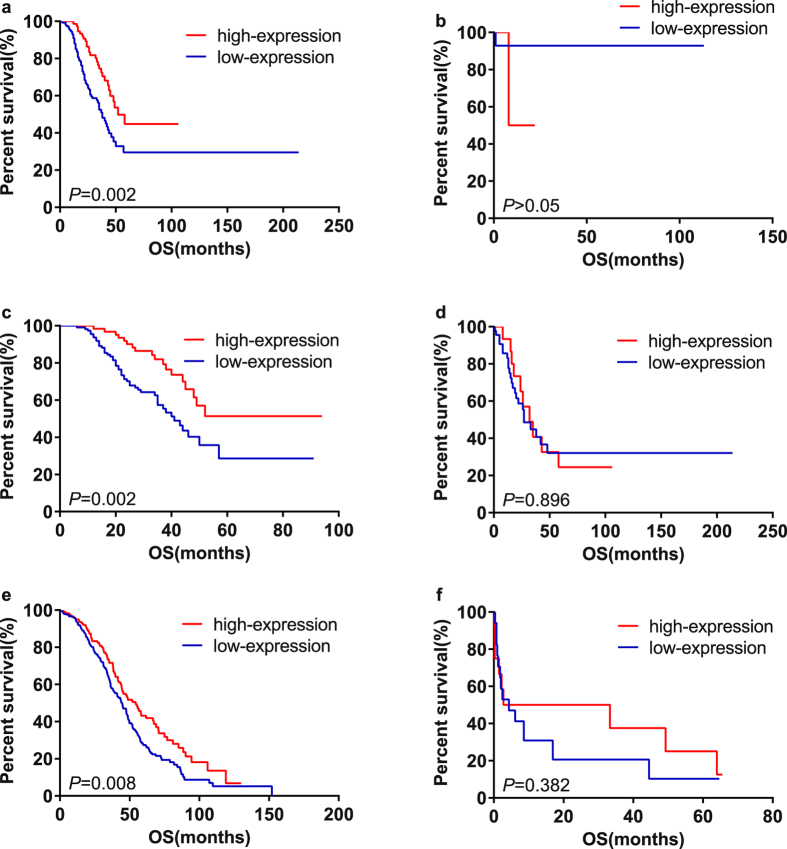
Stratification analysis of IL-6R mRNA on survival. (**a**) Kaplan-Meier analysis of patients with platinum treatment in dataset GSE9891. (**b**) Kaplan-Meier analysis of patients with no platinum treatment in dataset GSE9891. (**c**) Kaplan-Meier analysis of patients with taxane treatment in dataset GSE9891. (**d**) Kaplan-Meier analysis of patients with no taxane treatment in dataset GSE9891. (**e**) Kaplan-Meier analysis of patients with additional radiation therapy dataset in TCGA. (**f**) Kaplan-Meier analysis of patients with no additional radiation therapy dataset in TCGA.

We hypothesized that the use of medications affects the effect of IL-6R. In this article, there are two datasets (GSE9891, TCGA) that embody clinical treatment information and the survival analysis results were listed in Supplementary Table S3 and Fig. 5. Interestingly, in patients who had additional chemotherapy treatment, high expression level of IL- 6 R mRNA seemed to have better survival (HR = 0.495; 95% CI = 0.317–0.772; *P* = 0.002 for platinum treatment in GSE9891; HR = 0.438; 95% CI = 0.254–0.755; *P* = 0.003 for taxane treatment in GSE9891; HR = 0.697; 95% CI = 0.533–0.911; *P* = 0.008 for postoperative chemotherapy treatment in TCGA). While in patients who had no chemotherapy treatment, IL-6R expression is not associated with prognosis. (*P* values were 0.210, 0.897 and 0.538, respectively.)

### Correlation between IL-6 and IL-6ST

The IL-6 receptor is a protein complex consisting of an alpha chain, IL-6R, and IL-6 signal transducer (IL-6ST). Relationship between the mRNA expression of IL-6 and IL-6R, the relationship between IL-6 and IL-6ST, and between IL-6R and IL-6ST were all analyzed in those eight datasets. Interestingly, there was no statistically significant association between IL-6 and IL-6R or between IL-6R and IL-6ST. However, in five datasets, IL-6 was significantly associated with IL-6ST. And more importantly, analysis of 6 studies showed a higher correlation between IL-6 and IL-6ST in those patients who had a higher expression level of IL-6R mRNA. Then we performed a meta-analysis exploring the correlation between IL-6 and IL-6ST depending on the expression level of IL-6R based on Fisher’s z transformation of correlations. The pooled correlation coefficient were 0.225 for all patients, 0.310 for patients who had higher IL-6R expression and 0.182 for patients who had lower IL-6R expression (shown in Table 2).

**Table 2:** The Correlation Coefficient between IL-6 and IL-6ST Depend on the Expression Level of IL-6R.

GEO datasets	All patients		Patients with IL6R expression in Top 25%		Patients with IL6R expression in bottom 25%	
	r	*P*-value	r	*P*-value	r	*P*-value
GSE9891	0.112	0.084	0.400	**0.001**	−0.058	0.661
GSE17260	0.305	**0.001**	0.413	**0.029**	0.119	0.546
GSE26193	0.112	0.250	0.264	0.182	0.097	0.629
GSE26712	0.264	**<0.001**	0.377	**0.009**	−0.039	0.797
GSE32062	0.417	**<0.001**	0.526	**<0.001**	0.304	**0.014**
GSE49997	0.246	**<0.001**	0.212	0.142	0.283	**0.049**
GSE63885	0.069	0.555	0.145	0.553	0.035	0.886
TCGA	0.186	**<0.001**	0.175	**0.038**	0.294	**<0.001**
Pooled r (95%CI)	0.225 (0.141,0.305)	**<0.001**	0.310 (0.220,0.395)	**<0.001**	0.182 (0.087,0.274)	**<0.001**

### Biological processes and pathway analysis

Functional enrichment analysis on IL-6R and the most related genes was performed (all those genes were shown in Supplementary Table S4). Supplementary Table S5 lists the top 10 biological processes and pathway for its enrichment. One of the most significant biological processes is inflammatory response (GO: 0006954, *P* = 1.18E-06). Results also showed that those genes enriched in immune response (GO: 0006955, *P* = 4.44E-04). GO: 0045087, innate immune response; GO: 0002504, antigen processing and presentation of peptide or polysaccharide antigen via MHC class II and GO: 0019882, antigen processing and presentation are all belong to immune response.

The most important pathway is antigen processing and presentation (hsa04612, *P* = 3.04E-09). As with the results of biological processes, pathway analysis also shows that these genes are involved in immune responses, eg. antigen processing and presentation (hsa04612, *P* = 3.04E-09), phagosome (hsa04145, *P* = 1.16E-04), graft-versus-host disease (hsa05332, *P* = 9.20E-05), allograft rejection (hsa05330, *P* = 1.45E-04). Signaling pathway of bacteria and viruses infections are also included.

## Discussion

Ovarian cancer is the most common and serious threat to women’s life and health. Cytokines and chemokines play an important role in the development and progression of ovarian cancer through various mechanisms. To study the TME of ovarian cancer and to explore the meaning on the biological behavior of ovarian cancer are of great importance for the clinical treatment of ovarian cancer.

IL-6/IL-6R signaling was proved to play a significant role in the progression of ovarian cancer. IL-6R mRNA encodes a subunit of the IL-6 receptor complex. IL-6 is a potent pleiotropic cytokine that regulates cell growth and differentiation and plays an important role in the immune response. It also has additional roles in a variety of other processes such as metabolism and embryonic development. Dysfunction of the complex regulatory cytokine network might lead to acute and chronic inflammation^5^, autoimmune diseases^27^ or neoplastic disorders^28^. IL-6R acts as a part of the receptor for interleukin 6. Enrichment analysis of IL-6R and its most relevant genes did show that the most significant biological processes were inflammatory response and immune response. Pathway analysis also showed that these genes were most involved in immune responses.

Although Rath *et al*.^11^ have reported that expression of IL-6R mRNA was found to be up-regulated in ovarian malignancies, no study has indicated the prognostic impact of IL-6R mRNA expression in ovarian cancer tissues. To our knowledge, this is the first report that reused the published raw data and showed that high level of IL-6R mRNA expression was an independent factor for ovarian cancer patients. And this was consistent with Wouters.M’s finding that a high expression of IL-6R protein in ovarian cancer tissue was related to a better DSS^19^. Simultaneously, GSE9891 and TCGA datasets contain important treatment information, therefore, we analyzed the clinical significance of IL-6R mRNA with or without relevant treatment. Interestingly, high level of IL-6R mRNA expression was also a protective factor for patients with platinum or taxane or postoperative chemotherapy treatment, which could indicate that high expression of IL-6R mRNA might improve the sensitivity of chemotherapeutic agents. Although there were no statistical significance of IL-6R mRNA expression level in patients without those treatments in our study, considering the small sample size (16, 60 and 29, respectively), whether there was an association between them should be confirmed by other data.

IL-6R is a part of the receptor for interleukin 6 which binds to IL-6 with low affinity, but does not transduce a signal^29^. IL-6ST is necessary for this signal activation. Analysis of those eight selected datasets revealed that there was no significant correlation between IL-6R and IL-6 or between IL-6R and IL-6ST. However, IL-6 was significantly associated with IL-6ST in five datasets. The pooled correlation coefficients for IL-6 and IL-6ST were 0.310 and 0.182 in patients with IL6R expression in top 25% and patients with IL6R expression in bottom 25%, indicating that the higher the expression of IL-6R, the higher the correlation between IL-6 and IL-6ST. So we deduce that the expression level of IL-6R may affect the binding of IL-6 and IL-6ST and may further affect the activation of downstream pathways, such as Jak-STAT signaling pathway, PI3K-Akt signaling pathway, TNF signaling pathway and etc.

However, some details need to be further refined. First, this study included only eight eligible datasets, which resulted in relatively insufficiency data in the subgroup analyses. Second, due to the lack of some pivotal clinical parameters, association between those parameters and IL-6R mRNA expression can’t be shown, such as metastasis, treatment and so on. Although we found that high level of IL-6R mRNA expression was a protective factor for patients with chemotherapy treatment, this result should be verified by other clinical randomized trials. Third, there are six publications that reported stage of all patients, but the number of patients varies greatly in each stage. Thus, we reported both univariate and multivariate analysis results in this paper.

In conclusion, our results showed that mRNA levels of IL-6R in ovarian cancer was associated with better prognosis and sensitivity to chemotherapy and can potentially be used as a prognostic marker for this cancer. Taking the limitation of our study into consideration, the results should be regarded cautiously. Further prospective studies available of pivotal parameters are needed to verify the prognosis value of IL-6R in ovarian cancer patients. And the process of translation from mRNA to protein, microRNA regulation and post-translational modification need further study.

## Materials and Methods

### Search strategy

Electronic databases were searched through GEO database (last update by January 10, 2017) using the keywords “ovarian cancer” (http://www.ncbi.nih.gov/geo). Database searching was carried out by two researchers independently (Min Yang and Lei Lan).

### Data extraction and quality assessment

Date from all 8 eligible datasets was abstracted independently by two authors, using information recorded as follows: first author’s surname, publication year, origin of population, sample number, tumor stage, follow-up period and clinic outcome. Seven microarray datasets together with TCGA which embody IL-6R mRNA expression and survival data are collected, HRs and 95% CIs were evaluated by Cox proportional hazards model.

The quality of eight eligible studies was assessed according to the Newcastle-Ottawa Quality Assessment Scale (NOS) by two researchers independently. The quality scores span from 0 to 9, and higher the score is, higher the quality is.

### Statistical analysis

For those public microarray data, gene expression was represented by metric variables. We use *Cutoff Finder* (http://molpath.charite.de/cutoff) to determine a cutoff point and stratify patients into two groups^30^. The range of IL-6R mRNA values for each data and the corresponding cutoff value were listed in Supplementary Table S6. HRs and 95% CIs were calculated to measure the effective prognostic value of expression of IL-6R mRNA in ovarian cancer patients. Heterogeneity of HR was appraised by using the Cochran Q and I^2^ test. A random-effect model (the DerSimonian-Laird method) was applied when *P* < 0.1 or I^2^ > 50%. When heterogeneity was absent, a fixed-effect model (the Mante-Haenszel method) was employed.

Publication bias was assessed by Begg’s rank correlation method and Egger’s weighted regression method. All *P* values were two tailed, and all analyses were carried out using STATA software package (version 12.0) (Stata Corp LP, College Station, TX, USA).

For each dataset, we calculated the correlation coefficient between IL-6R and the remaining genes, and then matched the coefficients in all the eight datasets. Genes with absolute correlation coefficient which were greater than 0.3 in three or more publications were extracted. 116 genes were included in subsequent analysis (Supplementary Table S4).

Functional enrichment analysis of IL-6R and its related genes allows the identification of biological processes or functions. In this study, Gene Ontology Consortium (http://www.geneontology.org/) was used to analyze gene enrichment and to explore the biological processes of gene enrichment. Pathway mapping of IL-6R was done on the Database for Annotation, Visualization and Integrated Discovery (DAVID) (https://david.ncifcrf.gov/summary.jsp).

## Electronic supplementary material

supplementary materials
