# Supplementary material for: High mRNA expression level of IL-6R was associated with better prognosis for patients with ovarian cancer: a pooled meta-analysis
Source: Sci Rep. 2017 Aug 18;7:8769. doi: 10.1038/s41598-017-09333-8 (PMC5562889; doi:10.1038/s41598-017-09333-8)
Supplement: Supplementary file 1 — supplementary materials [file 41598_2017_9333_MOESM1_ESM.pdf]

High mRNA expression level of IL-6R was associated with better prognosis for patients with ovarian cancer： a pooled meta-analysis

Qu Chen[1#], Bin Xu[2,3#], Lei Lan[1], Da Yang[5], Min Yang[4], Jingting Jiang[2,3], Binfeng Lu[4], Yueping Shen[1, *]

Supplementary materials

Supplementary Figure S1 Funnel plots of publication biases on the relationships between IL-6R mRNA expression and overall survival in ovarian cancer patients.

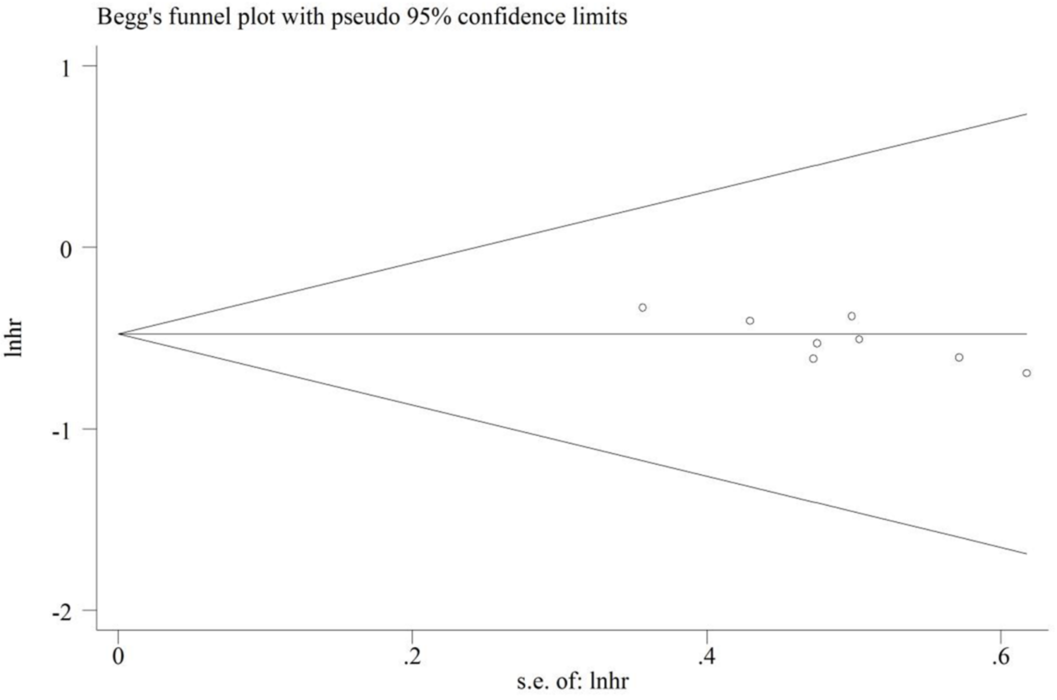

Supplementary Table S1 Survival Analysis of All Datasets

Datasets	variable	univariate analysis				multivariate analysis			
		HR	LCI	UCI	*P*-value	HR	LCI	UCI	*P*-value
GSE9891	stage	3.816	1.208	12.053	**0.022**	4.380	1.382	13.880	**0.012**
	taxane	0.568	0.373	0.864	**0.008**	0.503	0.328	0.772	**0.002**
	platinum	2.111	0.520	8.565	0.296	3.451	0.826	14.422	0.090
	age	1.424	0.960	2.112	0.079	1.379	0.927	2.052	0.113
	IL6R	0.542	0.350	0.840	**0.006**	0.527	0.339	0.820	**0.005**
GSE17260	stage	1.291	0.636	2.620	0.479	1.180	0.579	2.408	0.648
	grade	1.942	1.076	3.505	**0.028**	1.904	1.048	3.458	**0.034**
	IL6R	0.545	0.287	1.034	0.063	0.551	0.290	1.049	0.070
GSE26193	stage	4.197	2.192	8.034	**<0.001**	5.234	2.618	10.463	**<0.001**
	grade	1.063	0.661	1.709	0.801	0.627	0.377	1.044	0.073
	IL6R	0.602	0.366	0.991	**0.046**	0.614	0.372	1.011	0.055
GSE26712	subtype	1.511	1.069	2.135	**0.019**	1.457	1.030	2.063	**0.034**
	IL6R	0.666	0.464	0.956	**0.028**	0.692	0.481	0.995	**0.047**
GSE32062	subtype	0.890	0.617	1.283	0.531	0.958	0.658	1.395	0.821
	surgery	2.006	1.358	2.961	**<0.001**	1.927	1.297	2.865	**0.001**
	stage	1.465	0.981	2.188	0.062	1.428	0.950	2.148	0.087
	IL6R	0.590	0.379	0.918	**0.019**	0.596	0.383	0.929	**0.022**
GSE49997	age	1.763	1.044	2.977	**0.034**	1.630	0.957	2.776	0.072
	subtype	3.676	1.973	6.850	**<0.001**	3.595	1.923	6.723	**<0.001**
	grade	2.168	1.061	4.429	**0.034**	2.169	1.059	4.443	**0.034**
	IL6R	0.500	0.237	1.058	0.070	0.490	0.221	1.088	0.080
GSE63885	stage	2.214	1.093	4.485	**0.027**	2.114	1.040	4.298	**0.039**
	grade	2.321	1.040	5.178	**0.040**	2.342	1.038	5.283	**0.040**
	IL6R	0.684	0.420	1.114	0.127	0.634	0.387	1.039	0.071
TCGA	stage	2.712	1.442	5.098	**0.002**	2.572	1.367	4.840	**0.003**
	age	1.347	1.071	1.695	**0.011**	1.301	1.031	1.640	**0.026**
	IL6R	0.717	0.559	0.920	**0.009**	0.757	0.588	0.973	**0.030**

HR=hazard ratio; LCI=lower 95% confidence interval; UCI=upper 95% confidence interval.

Supplementary Table S2 Hazard Ratios of IL-6R mRNA for OS

GEO datasets	univariate analysis				multivariate analysis			
	HR	LCI	UCI	*P*-value	HR	LCI	UCI	*P*-value
GSE9891[*]	0.542	0.350	0.840	**0.006**	0.527	0.339	0.82	**0.005**
GSE17260[†]	0.545	0.287	1.034	0.063	0.551	0.290	1.049	0.070
GSE26193[‡]	0.602	0.366	0.991	**0.046**	0.614	0.372	1.011	0.055
GSE26712[#]	0.666	0.464	0.956	**0.028**	0.692	0.481	0.995	**0.047**
GSE32062[&]	0.590	0.379	0.918	**0.019**	0.596	0.383	0.929	**0.022**
GSE49997[§]	0.5	0.237	1.058	0.070	0.49	0.221	1.088	0.080
GSE63885[¶]	0.684	0.420	1.114	0.127	0.634	0.387	1.039	0.071
TCGA[※]	0.717	0.559	0.920	**0.009**	0.757	0.588	0.973	**0.030**

Multivariate analysis adjusted variables: [*]： Platinum (Yes vs No), Taxane(Yes vs No), Age(≥60 vs <60), Stage; [†]: Stage, Grade; [‡]:Stage, Grade; [#]: Subtype; [&]:Subtype, Surgery, Stage; [§]:Age(≥60 vs <60), Subtype, Grade; [¶]:Stage, Grade; [※]:Age(≥65 vs <65), Stage.

HR=hazard ratio; LCI=lower 95% confidence interval; UCI=upper 95% confidence interval.

Supplementary Table S3 Stratification Analysis of IL6R mRNA on Survival

GEO datasets	treatment	*N*	HR	LCI	UCI	*P*-value
GSE9891	Platinum-YES	224	0.495	0.317	0.772	**0.002**
GSE9891	Platinum -NO	16	5.916	0.366	95.516	0.210
GSE9891	Taxane-YES	180	0.438	0.254	0.755	**0.003**
GSE9891	Taxane -NO	60	0.952	0.452	2.003	0.897
TCGA	Postoperative chemotherapy treatment-YES	516	0.697	0.533	0.911	**0.008**
TCGA	Postoperative chemotherapy treatment -NO	29	0.760	0.316	1.826	0.538

N=sample size; HR=hazard ratio; LCI=lower 95% confidence interval; UCI=upper 95% confidence interval.

Supplementary Table S4 Correlation Coefficient of IL-6R

Gene	GSE9891	GSE17260	GSE26193	GSE26712	GSE32062	GSE49997	GSE63885	TCGA
IL6R	1	1	1	1	1	1	1	1
GMPR	0.382	0.358	0.366	0.265	0.387	-0.212	0.468	0.368
HOXD1	0.015	0.458	0.539	0.432	0.49	0.031	0.475	0.348
HPSE	0.359	0.331	0.414	0.275	0.454	0.226	0.304	0.317
RTP4	0.37	0.42	0.38	0.117	0.426	-	0.311	0.317
SMAD1	0.303	0.229	0.291	0.316	0.354	0.364	0.363	0.347
CHODL	0.3	0.423	0.407	0.149	0.339	-0.065	0.346	0.31
ELF4	0.309	0.196	0.32	0.335	0.296	-0.018	0.31	0.319
IFI16	0.315	0.389	0.294	0.125	0.425	-0.286	0.338	0.321
IRF1	0.448	0.305	0.404	0.213	0.522	0.03	0.257	0.358
LOC100506798	0.347	0.321	0.433	-	0.348	-	0.349	-
LOC401022	0.428	0.509	0.526	-	0.435	-	0.497	-
MTX2	0.293	0.374	0.48	0.061	0.314	0.338	0.364	0.173
NPR1	0.255	0.318	0.364	0.372	0.357	0.022	0.197	0.313
OTUD4	0.198	0.322	0.331	0.232	0.47	-	0.446	0.343
AIFM1	0.374	0.252	0.277	0.233	0.322	-	0.432	0.331
BTN3A3	0.311	0.288	0.32	0.092	0.345	0.094	0.253	0.307
CCDC109B	0.327	0.276	0.269	0.06	0.418	-	0.365	0.364
CFB	0.343	0.371	0.296	0.2	0.487	-	0.321	0.272
CXCL10	0.417	0.301	0.32	0.054	0.484	-0.14	0.281	0.28
CXCL11	0.388	0.266	0.371	0.029	0.444	-0.052	0.262	0.301
DAPL1	0.311	0.366	0.354	-	0.335	-	0.267	-

DENND2D	0.388	0.455	0.218	0.198	0.454	-	0.346	0.213
DTX3L	0.38	0.302	0.255	-	0.453	0.023	0.372	-
GBP2	0.39	0.23	0.35	0.191	0.364	0.248	0.159	0.338
GJB1	0.31	0.359	0.247	0.331	0.369	-0.018	0.257	0.294
HOXD4	0.208	0.35	0.319	-	0.388	0.023	0.431	-
IFIH1	0.15	0.362	0.286	0.055	0.44	0.348	0.268	0.31
LAP3	0.326	0.303	0.243	0.016	0.371	0.396	0.267	0.255
MUC1	0.329	0.284	0.33	0.311	0.43	-	0.172	0.255
NMI	0.337	0.224	0.327	0	0.371	0.456	0.226	0.28
NOD2	0.346	0.303	0.326	0.177	0.404	-	0.112	0.234
NOP16	0.338	0.315	0.365	0.223	0.324	-	0.276	-
NR3C1	0.355	0.306	0.394	-0.096	0.372	0.209	0.284	0.284
RGS14	0.189	0.37	0.324	0.06	0.409	-0.204	0.389	0.2
SP110	0.284	0.306	0.136	0.262	0.393	-0.153	0.346	0.322
TAP2	0.247	0.227	0.337	0.144	0.363	0.279	0.328	0.303
TAPBP	0.141	0.283	0.379	0.283	0.435	-0.079	0.37	0.308
TCIRG1	0.347	0.283	0.149	0.332	0.375	0.328	0.132	0.257
TM4SF1	0.293	0.352	0.345	0.102	0.462	-0.063	0.308	0.266
TNFAIP8	0.429	0.264	0.312	0.13	0.407	-0.23	0.178	0.362
UBA7	0.331	0.302	0.265	0.228	0.345	-	0.301	-
UBE2L6	0.308	0.252	0.301	0.172	0.377	0.146	0.322	0.227
AADAC	0.21	0.449	0.337	-0.038	0.434	0.057	0.156	0.194
ACPP	0.132	0.341	0.343	0.181	0.301	-0.051	0.128	0.16
ADRBK2	0.415	0.289	0.275	0.298	0.414	0.046	0.406	0.297

ANKRD6	0.156	0.231	0.325	0.182	0.351	-	0.216	0.321
ARHGAP26	0.459	0.294	0.204	0.127	0.382	0.032	0.158	0.364
BCL2L14	-0.039	0.355	-0.058	0.079	0.334	0.448	0	0.096
C2orf88	0.317	0.293	0.337	-	0.348	-	0.248	-
C5orf56	0.302	0.127	0.407	-	0.313	-	0.258	-
CALHM2	0.303	0.274	0.387	0.132	0.285	-	0.372	-
CD69	0.343	0.189	0.124	-0.084	0.409	0.717	0.067	0.248
CEBPD	0.182	0.301	0.053	0.081	0.32	0.347	-0.073	0.232
CFLAR	0.135	0.301	0.455	0.206	0.356	-0.193	0.199	0.247
CHI3L1	0.312	0.507	0.288	0.123	0.481	-0.175	0.223	0.276
CSF1	0.14	0.184	0.18	0.245	0.377	0.137	0.346	0.301
CXorf38	0.338	0.257	0.246	-	0.443	-0.145	0.353	-
CYP4B1	0.098	0.364	0.31	0.236	0.458	0.143	0.241	0.293
DCAF8	0.062	0.325	0.404	0.193	0.317	-	0.182	-
FAM65B	0.254	0.23	0.33	0.081	0.368	-	0.477	-
FGD2	0.301	0.147	0.359	0.111	0.37	0.125	0.069	0.168
FLJ43663	0.204	0.353	0.357	-	0.366	-	0.01	-
GBP1	0.318	0.169	0.264	0.113	0.372	0.113	0.124	0.327
GPD2	0.206	0.367	0.339	0.184	0.318	0.043	0.116	0.207
GPSM3	0.371	0.134	0.213	0.211	0.353	-0.289	0.316	0.216
GSDMD	0.356	0.052	0.297	0.243	0.307	-	0.386	-
HLA-DOB	0.043	0.189	0.326	0.058	0.394	0.121	0.317	0.265
HLA-DPB1	0.311	0.228	0.316	0.146	0.339	-	0.255	0.256
HLA-DRB1	0.31	0.287	0.343	0.226	0.376	-0.189	0.264	0.272

HLA-E	0.338	0.224	0.323	0.213	0.3	-0.251	0.33	0.275
HOXD3	0.355	0.299	0.363	0.192	0.372	0.036	0.239	0.214
IL15RA	0.329	0.257	0.282	0.294	0.362	0.13	0.21	0.303
IL4I1	0.372	0.332	0.299	-	0.469	-0.099	0.205	-
KIAA0494	0.182	0.501	0.223	0.165	0.352	0.584	0.169	0.283
KLK7	0.221	0.364	0.419	0.279	0.438	0.062	0.19	0.287
LGALS9	0.338	0.347	0.109	0.23	0.362	-0.042	0.288	0.279
LOC100506783	0.32	-	0.399	-	-	-	0.349	-
LOC100507463	0.352	-	0.322	-	-	-	0.333	-
LOC643201	0.209	0.203	0.44	-	0.356	-	0.385	-
LOC645431	0.262	0.34	0.394	-	0.419	-	0.248	-
LPAR3	0.178	0.287	0.326	0.265	0.426	-	0.32	-
LYN	0.343	0.333	0.05	0.098	0.402	0.15	0.152	0.276
MAT2B	0.208	0.08	0.335	-0.168	0.374	0.177	0.356	0.137
MID1IP1	0.149	0.412	0.048	0.179	0.311	0.059	0.313	0.182
MKNK1	0.135	0.367	0.264	0.081	0.312	0.546	0.213	0.216
MMAA	0.317	0.282	0.183	-	0.373	0.153	0.332	-
MPP7	0.101	0.343	0.367	-	0.38	-0.164	0.246	-
MTM1	0.295	0.269	0.36	0.214	0.412	-0.176	0.287	0.344
N4BP2L1	0.046	0.306	0.239	-0.06	0.305	-	0.352	-
OPLAH	0.203	0.245	0.367	0.141	0.339	0.226	0.35	0.111
PARP12	0.312	0.358	0.267	0.142	0.391	-	0.212	0.215
PARP14	0.35	0.33	0.238	-	0.439	-0.083	0.278	-
PARP3	0.27	0.127	0.305	0.059	0.325	-0.044	0.348	0.207

PARP9	0.332	0.319	0.164	-	0.438	-0.299	0.299	-
PLEK	0.314	0.176	0.151	0.097	0.345	0.33	0.164	0.179
PPARGC1B	0.214	0.253	0.438	-	0.402	0.066	0.325	-
PSME2	0.435	0.235	0.367	0.114	0.352	-0.058	0.274	0.256
PTPLAD2	0.33	0.308	0.259	-	0.348	-	0.146	-
RHBDF2	0.363	0.11	0.202	0.334	0.377	-	0.174	0.284
SAP30L	0.329	0.091	0.134	0.057	0.129	0.305	0.344	0.274
SCO2	0.353	0.249	0.156	0.044	0.361	0.445	0.185	0.099
SLC5A1	0.328	0.393	0.069	0.189	0.417	0.092	0.256	0.22
SQRDL	0.373	0.309	0.139	0.041	0.32	-0.243	0.212	0.233
TAPBPL	0.296	0.233	0.315	0.222	0.363	0.33	0.162	0.2
TLR3	0.315	0.27	0.29	-0.072	0.348	0.081	0.276	0.302
TNFRSF1B	0.404	0.221	0.19	0.181	0.381	0.696	0.21	0.24
TRIM14	0.237	0.354	0.115	0.281	0.411	-0.013	0.349	0.268
USP18	0.294	0.293	0.328	0.135	0.391	0.063	0.412	0.286
USP53	0.262	0.343	0.291	0.179	0.393	-	0.353	-
USP9X	0.226	0.31	0.19	0.092	0.31	-0.271	0.42	0.257
VNN3	0.046	0.412	0.008	-0.04	0.32	0.356	-0.028	0.113
WISP3	0.212	0.329	0.318	0.196	0.364	-0.019	0.142	0.227
WT1	0.242	0.356	0.364	0.204	0.388	0.2	0.294	0.278
XAF1	0.265	0.272	0.338	0.257	0.368	-	0.292	0.308
ZNF503	0.247	0.154	0.337	-	0.319	-0.166	0.312	-

Supplementary Table S5 the Top10 Biological Processes and Pathway of IL-6R and Related Genes

Analysis	ID	Biological processes	*P*-value	Genes
Biological Processes	GO:0006954	inflammatory response	1.18E-06	APOL3, TNFRSF1B, NMI, LYN, CSF1, GSDMD, CHI3L1, TLR3, NFKB1, SMAD1, IFI16, CXCL11, LGALS9, CXCL10
	GO:0060333	interferon-gamma-mediated signaling pathway	6.98E-06	NMI, HLA-DRB1, IRF1, HLA-DPB1, HLA-E, GBP2, GBP1
	GO:0045087	innate immune response	2.49E-05	IFIH1, NOD2, LYN, ELF4, CSF1, GSDMD, TRIM14, TLR3, NFKB1, IFI16, C2, HLA-E, MX2
	GO:0060337	type I interferon signaling pathway	6.17E-05	IRF1, XAF1, HLA-E, MX2, GBP2, PSMB8
	GO:0002504	antigen processing and presentation of peptide or polysaccharide antigen via MHC class II	1.74E-04	HLA-DRB1, HLA-DPB1, HLA-DMA, HLA-DOB
	GO:0002690	positive regulation of leukocyte chemotaxis	2.08E-04	GPSM3, IL6R, CXCL11, CXCL10
	GO:0050852	T cell receptor signaling pathway	4.27E-04	HLA-DRB1, PSME1, PSME2, NCK1, NFKB1, HLA-DPB1, PSMB8
	GO:0006955	immune response	4.44E-04	TNFRSF1B, HLA-DRB1, CTSS, HLA-DPB1, CXCL11, HLA-E, HLA-DMA, HLA-DOB, GBP2,

				TAPBP, CXCL10
	GO:0019882	antigen processing and presentation	4.60E-04	HLA-DRB1, CTSS, HLA-DPB1, HLA-E, PSMB8
	GO:0051092	positive regulation of NF-kappaB transcription factor activity	1.82E-03	CFLAR, NOD2, TRIM14, TLR3, NFKB1, LGALS9
Pathway	hsa04612	Antigen processing and presentation	3.04E-09	HLA-DRB1, PSME1, PSME2, TAP2, CTSS, HLA-DPB1, HLA-E, HLA-DMA, HLA-DOB, TAPBP
	hsa05150	Staphylococcus aureus infection	4.32E-05	HLA-DRB1, CFB, C2, HLA-DPB1, HLA-DMA, HLA-DOB
	hsa05168	Herpes simplex infection	4.99E-05	IFIH1, HLA-DRB1, TAP2, TLR3, NFKB1, HLA-DPB1, HLA-E, HLA-DMA, HLA-DOB
	hsa05332	Graft-versus-host disease	9.20E-05	HLA-DRB1, HLA-DPB1, HLA-E, HLA-DMA, HLA-DOB
	hsa05321	Inflammatory bowel disease (IBD)	9.84E-05	NOD2, HLA-DRB1, NFKB1, HLA-DPB1, HLA-DMA, HLA-DOB
	hsa04145	Phagosome	1.16E-04	TCIRG1, HLA-DRB1, TAP2, CTSS, HLA-DPB1, HLA-E, HLA-DMA, HLA-DOB
	hsa05330	Allograft rejection	1.45E-04	HLA-DRB1, HLA-DPB1, HLA-E, HLA-DMA,

			HLA-DOB
hsa04940	Type I diabetes mellitus	2.40E-04	HLA-DRB1, HLA-DPB1, HLA-E, HLA-DMA, HLA-DOB
hsa05164	Influenza A	2.59E-04	IFIH1, HLA-DRB1, TLR3, NFKB1, HLA-DPB1, HLA-DMA, HLA-DOB, CXCL10
hsa05152	Tuberculosis	2.87E-04	TCIRG1, NOD2, HLA-DRB1, NFKB1, CTSS, HLA-DPB1, HLA-DMA, HLA-DOB

Supplementary Table S6 Cutoff Value of IL-6R

GEO datasets	min	P_{25}	median	mean	P_{75}	max	cutoff value
GSE9891	3.321	5.119	6.016	5.979	6.820	8.489	6.487
GSE17260	-4.325	-0.683	0.000	-0.039	0.750	1.956	-0.559
GSE26193	3.713	5.281	5.744	5.873	6.524	8.875	6.349
GSE26712	3.639	3.996	4.183	4.224	4.403	5.169	4.257
GSE32062	-4.855	-0.347	0.334	0.263	0.944	3.150	-0.680
GSE49997	-1.444	0.151	0.664	0.732	1.149	4.096	1.265
GSE63885	5.356	6.463	6.972	6.999	7.573	9.195	6.802
TCGA	3.438	4.029	4.413	4.474	4.809	6.978	4.656
